# Low probability of intercept-based adaptive radar waveform optimization in signal-dependent clutter for joint radar and cellular communication systems

**DOI:** 10.1186/s13634-016-0411-6

**Published:** 2016-10-28

**Authors:** Chenguang Shi, Sana Salous, Fei Wang, Jianjiang Zhou

**Affiliations:** Key Laboratory of Radar Imaging and Microwave Photonics, Ministry of Education, Nanjing University of Aeronautics and Astronautics, Nanjing, 210016 China

**Keywords:** Low probability of intercept (LPI), Radar waveform optimization, Orthogonal frequency division multiplexing (OFDM), Multicarrier radar, Cellular communication systems

## Abstract

In this paper, we investigate the problem of low probability of intercept (LPI)-based adaptive radar waveform optimization in signal-dependent clutter for joint radar and cellular communication systems, where the radar system optimizes the transmitted waveform such that the interference caused to the cellular communication systems is strictly controlled. Assuming that the precise knowledge of the target spectra, the power spectral densities (PSDs) of signal-dependent clutters, the propagation losses of corresponding channels and the communication signals is known by the radar, three different LPI based criteria for radar waveform optimization are proposed to minimize the total transmitted power of the radar system by optimizing the multicarrier radar waveform with a predefined signal-to-interference-plus-noise ratio (SINR) constraint and a minimum required capacity for the cellular communication systems. These criteria differ in the way the communication signals scattered off the target are considered in the radar waveform design: (1) as useful energy, (2) as interference or (3) ignored altogether. The resulting problems are solved analytically and their solutions represent the optimum power allocation for each subcarrier in the multicarrier radar waveform. We show with numerical results that the LPI performance of the radar system can be significantly improved by exploiting the scattered echoes off the target due to cellular communication signals received at the radar receiver.

## Introduction

### Background and related works

The increasing demand for radio frequency (RF) spectrum has pushed for new techniques that allow for a flexible and shared use of spectrum among different radar and wireless communications systems, which has been attracting the interest of many scientists and engineers for the last decades [1–4]. As such, joint radar and wireless communication system is considered as a coexistence solution to the RF spectrum congestion problem, due to services with high bandwidth requirements and the exponential increase in the number of wireless devices [5]. In such joint systems, the radar and communication systems operate in the same bandwidth, without causing too much interference to each other.

Due to many advantages over single carrier waveforms in radar systems, such as frequency diversity, short time on the target and agile waveform optimization, multicarrier waveforms have been considered to be amongst the best candidates for radar systems. Reference [6] introduces a generalized multicarrier radar model, and novel multicarrier spread spectrum waveforms are proposed and generated utilizing the derived model. It is shown that such waveform outperforms well-known existing multicarrier waveforms, which can lower the peak to average power ratio. In [7] and [8], it is stated that orthogonal frequency division multiplexing (OFDM) radar signals offer a better range and Doppler resolution than other radar signals. Motivated by the recent interest in multicarrier waveforms for radar systems, [9] develops a new mechanism for spectrum sharing between radar and OFDM communication systems, where the authors optimize the OFDM waveforms for radar and communication systems by appropriately allocating the subcarriers based on the importance of each channel. Bica et al. in [10] propose radar waveform design algorithms in spectrum sharing environments based on two different applications, target characterization and target detection, where the communication signals scattered off the target are considered as interference in the objective functions. The work is further extended in [11] where the communication signals scattered off the target can be exploited at the radar receiver, which significantly improves its target detection performance. In [12], the methods of coexistence between radar and communications systems are studied, where the authors derive achievable bounds on performance for a receiver that observes communications and radar return in the same frequency band. A new spectrum sharing system architecture and set of coexistence mechanisms that mitigate RF interference effects on the exchange of internal state information between radar and communications systems are investigated in [13], which shrink the mimimum required standoff range between systems while sustaining the performance of each system. Overall, the previous studies lay a solid foundation for the problem of spectrum sharing in joint radar and communication systems, and it should be noted that the target detection performance can be improved by optimizing the radar transmission waveform while guaranteeing the quality of communication links.

The study of low probability of intercept (LPI) optimization in radar system has received sizeable impetus in recent years, due to its improvement for military operations in modern electronic warfare. A LPI radar is defined as a radar that utilizes a special emitted waveform intended to prevent a non-cooperative intercept receiver from intercepting and detecting its emission [14]. In order to achieve better LPI performance, it is necessary to adaptively control the radiation of the radar system. Transmit power and dwell time management, pulse compression, ultra-low side lobe antenna, and waveform agility are widely used to guarantee the LPI requirement. Thus, extensive research has been conducted in LPI optimization from various perspectives [15–28]. In [18], the authors propose an optimal sensor selection strategy based on passive sensor cooperation, where the results are extended in [20] and a time difference of arrival cooperation based radar radiation control in multiple aircraft platforms is proposed. Shi et al. address the LPI optimization schemes in radar networks for the first time [21–24], and it has been demonstrated that radar network systems with multiple transmitters and multichannel receivers can provide significant LPI performance advantages over traditional monostatic radar systems. In [25], the problem of robust waveform design for distributed multiple-radar systems based on LPI is studied, in which the true target spectrum is presumed to lie within an uncertainty class bounded by known upper and lower bounds. Simulation results are provided to show that the proposed robust waveform optimization methods are effective in enhancing the LPI performance of the distributed multiple-radar systems in the worst possible scenario. The work in [26–28] investigates the sensor scheduling algorithm of selecting and assigning sensors dynamically for target tracking, which can obtain a good tradeoff between the target tracking accuracy and the LPI performance. The authors in [29] propose a novel resource scheduling algorithm of the radar network system for target tracking in clutter. The relationship model between radar resources and target tracking accuracy is built, and then the sampling interval, transmit power, and waveform parameters are selected for better LPI performance and tracking accuracy.

### Motivation and major contributions

Generally speaking, all previous studies on the coexistence between radar and communication systems aim at improving the target detection performance of the radar system as well as enhancing the quality of service in communication systems. However, to the best of our knowledge, the problem of LPI based adaptive radar waveform optimization in signal-dependent clutter for joint radar and cellular communication systems, which has not been considered, needs to be investigated. Hence, the focus of this paper is on the problem of LPI-based radar waveform design for spectrum sharing, in which the radar adaptively optimizes the transmission waveform such that the interference caused to the cellular communications systems is strictly controlled. To this end, the total transmitted power of the radar system is minimized to improve its LPI performance. More specifically, our major contributions are listed as follows:

(a) By incorporating the radar transmitted signals, the communication signals, the target spectra, the power spectral densities (PSDs) of signal-dependent clutters, and the propagation losses of corresponding channels into the system model, various signal-to-interference-plus-noise ratios (SINRs) are derived to provide metrics for the target detection performance in the joint radar and cellular communication systems. These expressions of SINRs differ in the way the communication signals scattered off the target are considered as useful energy, as interference or ignored altogether at the radar receiver.

(b) The problem of LPI-based multicarrier OFDM radar waveform optimization in signal-dependent clutter for joint radar and cellular communication systems is studied. We first assume that the radar knows the exact perfect knowledge of the target spectra, the clutter PSDs, the propagation losses of corresponding channels, and the communication signals either by sensing with a spectrum analyzer or provided by the cellular communication systems. Then, different LPI-based criteria for radar waveform optimization are proposed, which minimize the total transmitted power of the radar system by optimizing the multicarrier radar waveform in signal-dependent clutter with a given SINR constraint and a minimum required capacity for the cellular communication systems.

(c) All the multicarrier radar waveform optimization criteria are formulated and proved to be convex, where the Karush-Kuhn-Tucker (KKT) conditions are derived to solve the resulting constrained problems.

(d) The numerical results demonstrate the importance of employing the communication signals scattered off the target for the improved LPI performance of the radar system via Monte-Carlo simulations. That is to say, the total transmitted power of the radar system can be reduced significantly by exploiting the scattering off the target due to communication signals for a predefined SINR threshold, which leads to better LPI performance to defend against the hostile passive intercept receivers.

The rest of this paper is organized as follows. The considered joint radar and cellular communication systems model is introduced in Section 2. In Section 3, the LPI-based radar waveform optimization criteria are proposed and the associated optimization problems are formulated and solved analytically. The performance of the proposed methods is validated by Monte Carlo simulations in Section 4. Finally, Section 5 concludes this paper.

*Notation:* The continuous time domain signal is denoted by *x*(*t*); *x*[*k*] is the associated sampled discrete time domain signal; and the frequency domain representation of the discrete sample *x*[*k*] is *X*[*k*]. A single lower case bold letter **x** represents a column vector with a given dimension. By *x*_*k*_, we denote the *k*th element of a vector **x**. The symbol ⊗ signifies the convolution operator. The superscript (·)^*T*^ and (·)^∗^ indicate transpose and optimality.

## Signal and system model

### Signal model

**Fig. 1:**
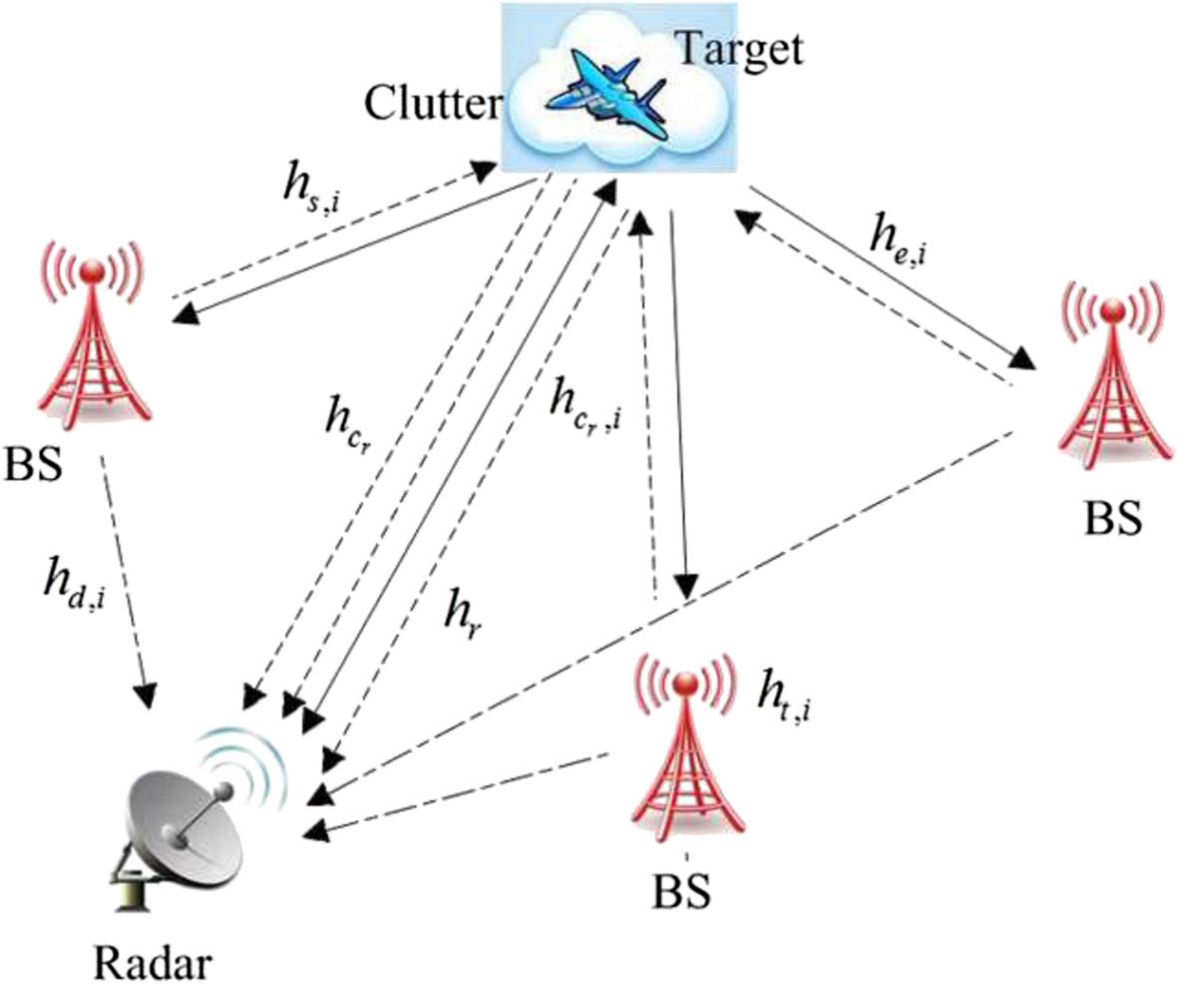
Joint radar and cellular communication systems model

The joint radar and cellular communication systems model is depicted in Fig. 1, which is composed of one monostatic radar and *N*_*t*_ communication base stations (BSs) with a goal to detect the target [11]. To increase the spectral efficiency, we consider the coexistence of radar and cellular communication systems in the same frequency band. It is assumed that both the radar and the cellular communication systems use multicarrier OFDM signals with *K* subcarriers, whose main advantage is separation of the frequency bandwidth into non-selective sub-bands [30]. The radar signal *x*_*r*_(*t*) can be given by [10]:

1 $$ x_{r}(t) = \sum\limits_{k = 0}^{K - 1} R_{k}e^{j2 \pi \left(f_{c} + k \Delta f\right)t},  $$

where *R*_*k*_ is the amplitude of the *k*th subcarrier for the radar signal, *f*_*c*_ is the carrier frequency, and *Δ* *f* is the subcarrier spacing. For the cellular communication systems, it is supposed that each of them utilizes a portion of the entire frequency bandwidth, that is, for ∀*i*∈{1,2,⋯,*N*_*t*_},

2 $$ x_{s, i}(t) = \sum\limits_{k \in \mathcal{F}_{i}} C_{i, k}e^{j2 \pi \left(f_{c} + k \Delta f\right)t},  $$

where $\mathcal {F}_{i} \subseteq \mathcal {F} = \{0, 1, \cdots, K-1\}$, and $\mathcal {F}$ denotes the set of equal spaced discrete frequencies representing the operational range of the joint radar and cellular communication systems. *C*_*i*,*k*_ is the amplitude of the *k*th subcarrier for the *i*th BS signal.

### System model

In Fig. 1, the radar receives the echo from the target due to the transmitted radar signals as well as the communication signals from the BSs, both scattered off the target and through a direct path. The cellular communications systems carry out their task of information transmission by broadcasting signals throughout the space. In addition, we assume that the radar antenna is directional and oriented towards the target, thus the target signal does not arrive at the cellular communication systems through a direct path, but only scattered off the target. For *N*_*t*_ communication BSs, the equation of the received signal at the radar system can be expressed in the continuous time domain as follows:

3 $$ y(t) = r(t)+\sum\limits_{i=1}^{N_{t}}[r_{s, i}(t)+s_{i}(t)+r_{c_{s},i}(t)]+r_{c_{r}}(t)+n(t),  $$

where *y*(*t*) represents the received signal at the radar receiver, *r*(*t*) is the echo from the target due to the radar transmitted signal, *r*_*s*,*i*_(*t*) is the scattering off the target due to the communication signal corresponding to the *i*th BS, *s*_*i*_(*t*) is the communication signal arriving through a direct line of sight path at the radar receiver corresponding to the *i*th BS. $r_{c_{r}}(t)$ denotes complex-valued, zero-mean Gaussian random process representing the signal-dependent clutter due to the radar transmitted signal. Likewise, $r_{c_{s},i}(t)$ is the complex-valued, zero-mean Gaussian random clutter due to the *i*th communication BS and *n*(*t*) stands for additive white Gaussian noise.

Here, the considered channels are given as follows: *h*_*r*_ for the radar-target-radar path, *h*_*e*,*i*_ for the radar-target-*i*th BS path, *h*_*s*,*i*_ for the *i*th BS-target-radar path, *h*_*d*,*i*_ for the *i*th direct BS-radar path, $h_{c_{r}}$ for the radar-clutter-radar path, $h_{c_{s}, i}$ for the *i*th BS-clutter-radar path, *h*_*t*,*i*_ for the communication inside the *i*th BS cell. The communication signal *x*_*s*,*i*_(*t*) is supposed to be deterministic and known at the radar receiver after a previous estimation step. We assume that the channels are stationary over the observation period. The channels *h*_*r*_(*t*), *h*_*s*,*i*_(*t*), $h_{c_{r}}(t)$, $h_{c_{s}, i}(t)$ and *h*_*e*,*i*_(*t*), corresponding to the target scattering, as well as the communication channels *h*_*d*,*i*_(*t*) and *h*_*t*,*i*_(*t*) are considered random and only known statistically. The channel impulse responses *h*_*r*_(*t*) and *h*_*s*,*i*_(*t*) are assumed to be zero-mean Gaussian random processes. Thus, for the cellular communication systems with *N*_*t*_ BSs, Eq. (3) is rewritten as:

4 $$ {{} {\begin{aligned} y(t) & = x_{r}(t) \otimes h_{r}(t)+\sum\limits_{i=1}^{N_{t}} \left[ x_{s, i}(t) \otimes h_{s, i}(t)+x_{s, i}(t) \otimes h_{d, i}(t) \right. \\ & \left. + x_{s, i}(t) \otimes h_{c_{s}, i}(t) \right] + x_{r}(t) \otimes h_{c_{r}}(t)+n(t). \end{aligned}}}  $$

Applying the Fourier transform to (4), we can have:

5 $$\begin{array}{*{20}l} Y(f) & = X_{r}(f) h_{r}(f)+\sum\limits_{i=1}^{N_{t}} \left\{ X_{s, i}(f) h_{s, i}(f)+X_{s, i}(f) h_{d, i}(f) \right. \\ & \left. + X_{s, i}(f) h_{c_{s}, i}(f) \right\} + X_{r}(f) h_{c_{r}}(f)+N(f). \end{array} $$

Then, the discrete signal spectrum can be equivalently written as:

6 $$ {{}{\begin{aligned} Y[k] & = X_{r}[k] h_{r}[k]+\sum\limits_{i=1}^{N_{t}} \left\{ X_{s, i}[k] h_{s, i}[k]+X_{s, i}[k] h_{d, i}[k] \right.\\ & \left. + X_{s, i}[k] h_{c_{s}, i}[k] \right\} + X_{r}[k] h_{c_{r}}[k]+N[k], \end{aligned}}}  $$

where $h_{r}[k] \sim \mathcal {CN}(0, \sigma _{h_{r}}^{2}[k])$, $h_{s, i}[k] \sim \mathcal {CN}(0, \sigma _{h_{s, i}}^{2}[k])$, and $N[k] \sim \mathcal {CN}(0, {\sigma _{n}^{2}}[k])$. $\sigma _{h_{r}}^{2}[k]$ and $\sigma _{h_{s, i}}^{2}[k]$ are the spectral variances of the corresponding channels for the *k*th subcarrier. ${\sigma _{n}^{2}}[k]$ is the variance of the noise. $P_{c_{r}}[k]$ and $P_{c_{s}, i}[k]$ denote the PSDs of the signal-dependent clutters due to the radar and cellular communication BS transmitted signals for the *k*th subcarrier, respectively.

Therefore, the probabilistic model of the received radar signal is:

7 $$\begin{array}{*{20}l} Y[k] \sim \mathcal{CN}(0, {\sigma_{Y}^{2}}[k]), \end{array} $$

where the spectral variance of *Y*[*k*] can be expressed as:

8 $$ {{} {\begin{aligned} {\sigma_{Y}^{2}}[k] & = |X_{r}[k]|^{2} \sigma_{h_{r}}^{2}[k] + \sum\limits_{i=1}^{N_{t}} \left\{ |X_{s, i}[k]|^{2}\sigma_{h_{s, i}}^{2}[k] + |X_{s, i}[k]|^{2}L_{d, i}[k] \right. \\ & \left. + |X_{s, i}[k]|^{2}P_{c_{s}, i}[k] L_{c_{s}, i}[k] \right\} + |X_{r}[k]|^{2} P_{c_{r}}[k]L_{c_{r}}[k] + {\sigma_{n}^{2}}[k], \end{aligned}}}  $$

where |*X*_*r*_[*k*]|^2^ and |*X*_*s*,*i*_[*k*]|^2^ are the power of the radar and the *i*th BS signals for the *k*th subcarrier, respectively. The propagation losses of the corresponding channels for the *k*th subcarrier are [30]:

9 $$ {{\left\{\begin{aligned} L_{d, i}[k] &= \frac{G_{s}G_{t}{\lambda_{k}^{2}}}{(4 \pi)^{2}d_{b, i}^{2}}, \\ L_{c_{s}, i}[k] &= \frac{G_{s}G_{t}{\lambda_{k}^{2}}}{(4 \pi)^{3}d_{s, i}^{2}{d_{r}^{2}}}, \\ L_{c_{r}}[k] &= \frac{{G_{t}^{2}}{\lambda_{k}^{2}}}{(4 \pi)^{3}{d_{r}^{4}}}, \end{aligned} \right.}}  $$

where *G*_*t*_ is the antenna gain of the radar, *G*_*s*_ is the antenna gain of the communication system, *λ*_*k*_ is the wavelength at *k*th subcarrier. *d*_*r*_, *d*_*s*,*i*_, and *d*_*b*,*i*_ represent the distances between the radar and the target, between the *i*th communication BS and the target, and between the radar and the *i*th BS, respectively.

## Problem formulation

In this section, we propose three different approaches to LPI-based adaptive radar waveform optimization for joint radar and cellular communication systems. It is assumed that the precise characteristics of the target spectra, the clutter PSDs, the propagation losses of corresponding channels, and the communication signals are known by the radar. Our goal here is to adaptively design the multicarrier radar waveform in signal-dependent clutter with a predefined target detection constraint and a minimum required capacity for the cellular communication systems that optimizes the LPI performance of the radar system. In this paper, various SINRs are derived as metrics for target detection performance in the joint radar and cellular communication systems, which differ in the way the communication signals scattered off the target are considered at the radar receiver: (1) as useful energy, (2) as interference, or (3) ignored altogether. Subsequently, we employ the presented LPI-based criteria to obtain the objective functions to be minimized in the optimization problems. The resulting optimization problems in this paper are solved analytically and their solutions represent the optimum power allocation for each subcarrier in the multicarrier radar waveform.

### LPI based radar waveform optimization Criterion 1

Here, the SINR is utilized as a metric for target detection performance in the joint radar and cellular communication systems. In this case, the scattered communication signals from the target are considered as useful energy at the radar receiver. Based on the derivations in [10, 11], the achievable SINR can be described as follows:

10 $$ {{\begin{aligned} \text{SINR} & \triangleq \sum\limits_{k=0}^{K-1} \frac{|X_{r}[k]|^{2} \sigma_{h_{r}}^{2}[k] + \sum_{i=1}^{N_{t}}|X_{s, i}[k]|^{2} \sigma_{h_{s, i}}^{2}[k]}{|X_{r}[k]|^{2} P_{c_{r}}[k]L_{c_{r}}[k] + \sum_{i=1}^{N_{t}} \left\{ |X_{s, i}[k]|^{2}L_{d, i}[k]+|X_{s, i}[k]|^{2}P_{c_{s}, i}[k] L_{c_{s}, i}[k] \right\} +{\sigma_{n}^{2}}[k]} \\ & = \sum\limits_{k=0}^{K-1} \frac{|X_{r}[k]|^{2} |H_{r}[k]|^{2}L_{r}[k]+\sum_{i=1}^{N_{t}}|X_{s, i}[k]|^{2}|H_{s, i}[k]|^{2}L_{s, i}[k]}{|X_{r}[k]|^{2} P_{c_{r}}[k]L_{c_{r}}[k]+\sum_{i=1}^{N_{t}} \left\{ |X_{s, i}[k]|^{2}L_{d, i}[k]+|X_{s, i}[k]|^{2}P_{c_{s}, i}[k] L_{c_{s}, i}[k] \right\} +{\sigma_{n}^{2}}[k]}. \end{aligned}}}  $$

It is worth pointing out that |*H*_*r*_[*k*]|^2^ *L*_*r*_[*k*] denotes the spectral variance of *h*_*r*_[*k*], i.e., $h_{r}[k] \sim \mathcal (0, |H_{r}[k]|^{2}L_{r}[k])$, where *H*_*r*_[*k*] is the target spectrum for the radar-target-radar path. Likewise, |*H*_*s*,*i*_[*k*]|^2^ *L*_*s*,*i*_[*k*] denotes the spectral variance of *h*_*s*,*i*_[*k*], i.e., $h_{s, i}[k] \sim \mathcal (0, |H_{s, i}[k]|^{2}L_{s, i}[k])$, where *H*_*s*,*i*_[*k*] is the target spectrum for the *i*th BS-target-radar path. The propagation losses of the corresponding channels for the *k*th subcarrier can be expressed as follows [30]:

11 $$ \left\{ \begin{aligned} L_{r}[k] &= \frac{{G_{t}^{2}}{\lambda_{k}^{2}}}{(4 \pi)^{3}{d_{r}^{4}}}, \\ L_{s, i}[k] &= \frac{G_{s}G_{t}{\lambda_{k}^{2}}}{(4 \pi)^{3}d_{s, i}^{2}{d_{r}^{2}}}. \end{aligned} \right.  $$

We can notice from (10) that the SINR is related to the radar transmission waveform, the communication waveform, the target spectra, the PSDs of signal-dependent clutters, and the propagation losses of corresponding channels.

In this paper, we concentrate on the LPI-based radar waveform optimization for the joint radar and cellular communication systems to minimize the radar transmitted power for a predefined target detection performance. We impose a minimum capacity constraint per channel for the cellular communication systems and for the radar system an upper bound on the transmit power per channel. Eventually, the adaptive radar waveform optimization based on LPI can be formulated as:

12a $$\min\limits_{\left\{|X_{r}[k]|^{2} \right\}}~\sum\limits_{k=0}^{K-1} |X_{r}[k]|^{2}, $$

12b $$ s.t.:\quad\left\{ \begin{array}{l} \text{SINR} \geq \text{SINR}_{\text{min}},\\ \text{log} \left (1+\frac{|X_{s, i}[k]|^{2}L_{t, i}[k]}{|X_{r}[k]|^{2}{|H_{e, i}[k]|}^{2}L_{e, i}[k] + {\sigma_{n}^{2}}[k]} \right) \geq t_{k},\\ 0 \leq |X_{r}[k]|^{2} \leq P_{\text{max},k}. \end{array} \right.  $$

where SINR_min_ denotes the predefined SINR threshold, which is the minimum SINR requirement for the radar to satisfy its basic target detection need. *t*_*k*_ is the minimum required capacity for the *k*th subcarrier in the communication system. The transmitted power for the *k*th subcarrier is constrained by a maximum value *P*_max,*k*_ and a minimum value 0. *L*_*t*,*i*_[*k*] and *L*_*e*,*i*_[*k*] are the propagation losses of the corresponding channels [30]:

13 $$ \left\{ \begin{aligned} L_{t, i}[k] &= \frac{{G_{s}^{2}}{\lambda_{k}^{2}}}{(4 \pi)^{2}d_{t, i}^{2}}, \\ L_{e, i}[k] &= \frac{G_{s}G_{t}{\lambda_{k}^{2}}}{(4 \pi)^{3}d_{s, i}^{2}{d_{r}^{2}}}, \end{aligned} \right.  $$

where *d*_*t*,*i*_ is the radius of the *i*th communication cell. Note that the left-hand side of the second constraint in (12b) denotes the channel capacity of the communication systems, which is related to the mutual information between the received signals at BSs and the transmitted communication signals. The minimum capacity constraint per channel for the communication systems must be maintained above a predetermined threshold *t*_*k*_ inside a cell [5], which guarantees the quality of communication links. The communication channel inside the cell is represented by the impulse response *h*_*t*,*i*_(*t*). The interference is represented by the radar signals that are scattered off the target and arrive inside the cell, whose spectrum is denoted by *H*_*e*,*i*_(*t*). After simplifying the constraints and using the notation *x*_*k*_=|*X*_*r*_[*k*]|^2^, we can rewrite the optimization problem in (12) as:

14a $$\min\limits_{\{x_{k}\}}~ \sum\limits_{k=0}^{K-1} x_{k}, $$

14b $$\begin{array}{*{20}l} &s.t.:\quad\left\{ \begin{array}{l} \sum_{k=0}^{K-1} \frac{x_{k}m_{k} + a_{k}}{x_{k}n_{k} + b_{k}} \geq \text{SINR}_{\text{min}},\\ \mathbf{0} \leq \mathbf{x} \leq \mathbf{d}. \end{array} \right. \end{array} $$

where we define $a_{k} = \sum _{i=1}^{N_{t}}|X_{s, i}[k]|^{2}|H_{s, i}[k]|^{2}L_{s, i}[k]\phantom {\dot {i}\!}$, $b_{k} = \sum _{i=1}^{N_{t}}\left [|X_{s, i}[k]|^{2}L_{d, i}[k]+|X_{s, i}[k]|^{2}P_{c_{s}, i}[k] L_{c_{s}, i}[k]\right ]+{\sigma _{n}^{2}}[k]\phantom {\dot {i}\!}$, *m*_*k*_=|*H*_*r*_[*k*]|^2^ *L*_*r*_[*k*], $n_{k} = P_{c_{r}}[k]L_{c_{r}}[k]\phantom {\dot {i}\!}$, and $d_{k} = \text {min}\left \{P_{\text {max},k}, \frac {1}{|H_{e, i}[k]|^{2}L_{e, i}[k]}\left [\frac {|X_{s, i}[k]|^{2} L{t, i}[k]}{e^{t_{k}}-1} - {\sigma _{n}^{2}}[k]\right ]\right \}$.

Since

15 $$\begin{array}{*{20}l} \frac{\partial}{\partial x_{k}} \left(\frac{x_{k}m_{k} + a_{k}}{x_{k}n_{k} + b_{k}} \right)= \frac{m_{k}b_{k} - n_{k}a_{k}}{(x_{k}n_{k} + b_{k})^{2}} > 0, \end{array} $$

16 $$\begin{array}{*{20}l} \frac{\partial^{2}}{\partial {x_{k}^{2}}} \left(\frac{x_{k}m_{k} + a_{k}}{x_{k}n_{k} + b_{k}} \right)= -2\frac{m_{k}b_{k} - n_{k}a_{k}}{(x_{k}n_{k} + b_{k})^{3}} < 0, \end{array} $$

it is shown that the SINR in (15) and (16) is increasing and concave with respect to *x*_*k*_. Hence, the first constraint in (14b) constitutes a convex feasible set over *x*_*k*_ for all subcarriers, while the objective function is affine and the power constraint **0**≤**x**≤**d** is the intersection of 2 *K* half-spaces. Therefore, the optimization problem (14) is convex.

Then, we define a modified convex minimization problem by the method of Lagrange multipliers, which can be employed to solve the constrained optimization problem in (14). The Lagrange of problem (14) can be equivalently expressed by:

17 $$\begin{array}{*{20}l} L(\mathbf{x}, \boldsymbol{\lambda}_{1}, \boldsymbol{\lambda}_{2},\lambda_{3}) & =\sum\limits_{k=0}^{K-1}x_{k}+\boldsymbol{\lambda}_{1}^{T}(-\mathbf{x})+\boldsymbol{\lambda}_{2}^{T}(\mathbf{x}-\mathbf{d}) \\ & + \lambda_{3} \left(\text{SINR}_{\text{min}} - \sum\limits_{k=0}^{K-1} \frac{x_{k} m_{k} + a_{k}}{x_{k} n_{k} + b_{k}} \right), \end{array} $$

where ***λ***_1_≥**0**, ***λ***_2_≥**0**, and *λ*_3_≥0 represent slack variables. Due to the convex nature of problem (14), the KKT conditions are the sufficient and necessary conditions for the global optimality of $x_{k}^{*}$. Therefore, the KKT conditions can be developed as follows:

18a $$\begin{array}{*{20}l} \frac{\partial L}{\partial x_{k}}=1-\lambda_{1,k}^{*}+\lambda_{2,k}^{*}-\lambda_{3}^{*} \cdot \frac{m_{k}b_{k} - n_{k}a_{k}}{(n_{k}x_{k}^{*}+b_{k})^{2}}=0, & \end{array} $$

18b $$\begin{array}{*{20}l} (\boldsymbol{\lambda}_{1}^{*})^{T}(-\mathbf{x}^{*}) = 0, & \end{array} $$

18c $$\begin{array}{*{20}l} (\boldsymbol{\lambda}_{2}^{*})^{T}(\mathbf{x}^{*}-\mathbf{d}) = 0, & \end{array} $$

18d $$\begin{array}{*{20}l} \lambda_{3} \cdot \left(\text{SINR}_{\text{min}} - \sum\limits_{k=0}^{K-1} \frac{x_{k}^{*} m_{k} + a_{k}}{x_{k}^{*} n_{k} + b_{k}} \right) = 0, & \end{array} $$

18e $$\begin{array}{*{20}l} 0 \leq x_{k}^{*} \leq d_{k}, & \end{array} $$

18f $$\begin{array}{*{20}l} \boldsymbol{\lambda}_{1}^{*} \geq \mathbf{0}, & \end{array} $$

18g $$\begin{array}{*{20}l} \boldsymbol{\lambda}_{2}^{*} \geq \mathbf{0}, & \end{array} $$

18h $$\begin{array}{*{20}l} \lambda_{3}^{*} \geq 0. & \end{array} $$

To be specific, if **x** ^∗^ is the optimal solution, it must satisfy the stationarity condition $\frac {\partial L}{\partial x_{k}}=0$, primal feasibility $\sum _{k=0}^{K-1} \frac {x_{k}^{*} m_{k} + a_{k}}{x_{k}^{*} n_{k} + b_{k}} \geq \text {SINR}_{\text {min}}$, **0**≤**x** ^∗^≤**d**, dual feasibility $\boldsymbol {\lambda }_{1}^{*} \geq \mathbf {0}$, $\boldsymbol {\lambda }_{2}^{*} \geq \mathbf {0}$, $\lambda _{3}^{*} \geq 0$ and complementary slackness which states that a primal constraint is satisfied with equality, if and only if, the associated dual variable is strictly greater than zero [10]. Thus, when **x** ^∗^ is optimal, we obtain:

19 $$\begin{array}{*{20}l} x_{k}^{*}=-\frac{b_{k}}{n_{k}}+\frac{1}{n_{k}}\sqrt{\frac{\lambda_{3}^{*}(m_{k}b_{k} - n_{k}a_{k})}{1-\lambda_{1,k}^{*}+\lambda_{2,k}^{*}}}. \end{array} $$

From (14) and (17), it can be seen that the optimality conditions can be separately investigated for three possibilities regarding the optimal allocated power in each subcarrier. At the optimality, each subcarrier can be allocated either with no power ($x_{k}^{*}=0$), with maximum transmitting power ($x_{k}^{*}=d_{k}$), or with power between these two extreme cases ($0<x_{k}^{*}<d_{k}$).

(a) If $0<x_{k}^{*}<d_{k}$, then $\lambda _{1,k}^{*}=\lambda _{2,k}^{*}=0$, we have:

20 $$\begin{array}{*{20}l} 0<-\frac{b_{k}}{n_{k}}+\frac{1}{n_{k}}\sqrt{\lambda_{3}^{*}\left(m_{k}b_{k} - n_{k}a_{k}\right)}<d_{k}\\ \Leftrightarrow \frac{{b_{k}^{2}}}{\lambda_{3}^{*}} < \left(m_{k}b_{k} - n_{k}a_{k}\right) < \frac{\left(n_{k}d_{k}+b_{k}\right)^{2}}{\lambda_{3}^{*}}. \end{array} $$

Then, $x_{k}^{*}$ can be computed as:

21 $$\begin{array}{*{20}l} x_{k}^{*}=-\frac{b_{k}}{n_{k}}+\frac{1}{n_{k}}\sqrt{\lambda_{3}^{*}(m_{k}b_{k} - n_{k}a_{k})}, \end{array} $$

where $\lambda _{3}^{*}$ is a constant determined by the SINR constraint:

22 $$\begin{array}{*{20}l} \sum\limits_{k=0}^{K-1} \frac{x_{k}^{*}m_{k} + a_{k}}{x_{k}^{*}n_{k} + b_{k}} \geq \text{SINR}_{\text{min}}. \end{array} $$

(b) If $x_{k}^{*}=0$, then $\lambda _{1,k}^{*}>0$, $\lambda _{2,k}^{*}=0$, we can obtain:

23 $$ {{} {\begin{aligned} x_{k}^{*}+\frac{b_{k}}{n_{k}}& =\frac{1}{n_{k}}\sqrt{\frac{\lambda_{3}^{*}(m_{k}b_{k} - n_{k}a_{k})}{1-\lambda_{1,k}^{*}}}>\frac{1}{n_{k}}\sqrt{\lambda_{3}^{*}(m_{k}b_{k} - n_{k}a_{k})}\\ & \Leftrightarrow (m_{k}b_{k} - n_{k}a_{k}) > \frac{{b_{k}^{2}}}{\lambda_{3}^{*}}. \end{aligned}}}  $$

Then, $x_{k}^{*}$ can be given by:

24 $$\begin{array}{*{20}l} x_{k}^{*}=0. \end{array} $$

(c) If $x_{k}^{*}=d_{k}$, then $\lambda _{1,k}^{*}=0$, $\lambda _{2,k}^{*}>0$, we can have:

25 $$ {{} {\begin{aligned} x_{k}^{*}+\frac{b_{k}}{n_{k}}& =\frac{1}{n_{k}}\sqrt{\frac{\lambda_{3}^{*}(m_{k}b_{k} - n_{k}a_{k})}{1+\lambda_{2,k}^{*}}}<\frac{1}{n_{k}}\sqrt{\lambda_{3}^{*}(m_{k}b_{k} - n_{k}a_{k})}\\ & \Leftrightarrow (m_{k}b_{k} - n_{k}a_{k}) > \frac{(n_{k}d_{k}+b_{k})^{2}}{\lambda_{3}^{*}}. \end{aligned}}}  $$

Then, $x_{k}^{*}$ is obtained as:

26 $$\begin{array}{*{20}l} x_{k}^{*}=d_{k}. \end{array} $$

Therefore, the optimal power allocation solution can be derived as follows:

27 $$ {{}\fontsize{8.4}{6} \begin{aligned} x_{k}^{*}=\left\{ \begin{array}{ll} 0,& (m_{k}b_{k} - n_{k}a_{k}) \leq \frac{{b_{k}^{2}}}{\lambda_{3}^{*}}, \\ -\frac{b_{k}}{n_{k}} \,+\, \frac{\sqrt{\lambda_{3}^{*}(m_{k}b_{k} - n_{k}a_{k})}}{n_{k}}, & \frac{{b_{k}^{2}}}{\lambda_{3}^{*}} < (m_{k}b_{k} - n_{k}a_{k}) < \frac{(n_{k}d_{k}+b_{k})^{2}}{\lambda_{3}^{*}},\\ d_{k},&(m_{k}b_{k} - n_{k}a_{k}) \geq \frac{(n_{k}d_{k}+b_{k})^{2}}{\lambda_{3}^{*}}, \end{array} \right. \end{aligned}}  $$

where $\lambda _{3}^{*}$ is the Lagrange dual variable corresponding to the constraint on the SINR performance:

28 $$ \sum\limits_{k=0}^{K-1} \frac{x_{k}^{*} m_{k} + a_{k}}{x_{k}^{*} n_{k} + b_{k}} \geq \text{SINR}_{\text{min}}.  $$

*Remark 1:* Note that our solution scheme is to choose $\lambda _{3}^{*}$ as a search variable, and use the results of (27) to identify the optimal power allocation for all the channels. The importance of the derived solution (27) lies in the fact that it provides an explicit relation between the power allocation in each subcarrier and the resulting value for $\lambda _{3}^{*}$. Criterion 1 defines a procedure which finally provides optimal transmitting power allocation, and consequently, the optimum LPI performance. The iterative procedure is listed in Algorithm 1.

*Remark 2:* As aforementioned, the expression of SINR in (10) is a function of the radar transmission waveform, the cellular communication waveforms, the target spectra, the PSDs of signal-dependent clutters, and the propagation losses of corresponding channels. Thus, the precise characteristics of the communication signals, the target spectra, the clutter PSDs, and the propagation losses of corresponding channels are required to obtain the optimal solution (27). In practice, the transmitted cellular communication signals and the target spectra can be provided by the communication systems and the target database based on the target-radar/BSs orientations, respectively [11, 25]. While the clutter PSDs and the propagation losses of corresponding channels can be obtained by sensing with a spectrum analyzer or propagation modeling [25].



### LPI-based radar waveform optimization Criterion 2

Next, if the communication signals scattered off the target are received at the radar receiver and considered as interference, the overall SINR can be written as:

29 $$ \text{SINR} \triangleq \sum\limits_{k=0}^{K-1} \frac{x_{k}m_{k}}{x_{k}n_{k} + a_{k} + b_{k}}.  $$

Similarly, the adaptive radar waveform optimization approach based on LPI can be given by:

30a $$\min\limits_{\{x_{k}\}}~\sum\limits_{k=0}^{K-1} x_{k}, $$

30b $$\begin{array}{*{20}l} &s.t.:\quad\left\{ \begin{array}{l} {\sum\nolimits}_{k=0}^{K-1} \frac{x_{k}m_{k}}{x_{k}n_{k} + a_{k} + b_{k}} \geq \text{SINR}_{\text{min}}, \\ \mathbf{0} \leq \mathbf{x} \leq \mathbf{d}. \end{array} \right. \end{array} $$

We invoke the Lagrange multiplier technique yielding an objective function:

31 $$ {{}{\begin{aligned} L(\mathbf{x}, \boldsymbol{\lambda}_{1}, \boldsymbol{\lambda}_{2},\lambda_{3}) & = \sum\limits_{k=0}^{K-1}x_{k}+ \boldsymbol{\lambda}_{1}^{T}(-\mathbf{x})+\boldsymbol{\lambda}_{2}^{T}(\mathbf{x}-\mathbf{d})\\ & + \lambda_{3} \left(\text{SINR}_{\text{min}} - \sum\limits_{k=0}^{K-1} \frac{x_{k} m_{k}}{x_{k} n_{k} + a_{k} + b_{k}} \right). \end{aligned}}}  $$

Taking the first derivative of (31) with respect to *x*_*k*_, and setting it to zero, we thus obtain:

32 $$\begin{array}{*{20}l} \frac{\partial L}{\partial x_{k}}=1-\lambda_{1,k}+\lambda_{2,k}-\lambda_{3} \cdot \frac{m_{k}(a_{k}+b_{k})}{(n_{k}x_{k}+a_{k}+b_{k})^{2}}=0. \end{array} $$

After basic algebraic manipulations, we can reach the optimal solution $x_{k}^{*}$ as a function of the Lagrange multipliers:

33 $$\begin{array}{*{20}l} x_{k}^{*}=-(\frac{a_{k}+b_{k}}{n_{k}})+\frac{1}{n_{k}}\sqrt{\frac{\lambda_{3}^{*}m_{k}(a_{k}+b_{k})}{1-\lambda_{1,k}^{*}+\lambda_{2,k}^{*}}}. \end{array} $$

From complementary slackness, we must consider the following three cases:

(a) If $0<x_{k}^{*}<d_{k}$, then $\lambda _{1,k}^{*}=\lambda _{2,k}^{*}=0$, we have:

34 $$\begin{array}{*{20}l} 0<-(\frac{a_{k}+b_{k}}{n_{k}})+\frac{1}{n_{k}}\sqrt{\lambda_{3}^{*}m_{k}(a_{k}+b_{k})}<d_{k}\\ \Leftrightarrow \frac{(a_{k} + b_{k})^{2}}{\lambda_{3}^{*}} < m_{k}(a_{k} + b_{k}) < \frac{(n_{k}d_{k} + a_{k} + b_{k})^{2}}{\lambda_{3}^{*}}. \end{array} $$

Then, $x_{k}^{*}$ can be derived as:

35 $$\begin{array}{*{20}l} x_{k}^{*}=-\left(\frac{a_{k}+b_{k}}{n_{k}}\right)+\frac{1}{n_{k}}\sqrt{\lambda_{3}^{*}m_{k}(a_{k} + b_{k})}, \end{array} $$

where $\lambda _{3}^{*}$ is a constant determined by the SINR constraint:

36 $$\begin{array}{*{20}l} \sum\limits_{k=0}^{K-1} \frac{x_{k}^{*} m_{k}}{x_{k}^{*} n_{k} + a_{k} + b_{k}} \geq \text{SINR}_{\text{min}}. \end{array} $$

(b) If $x_{k}^{*}=0$, then $\lambda _{1,k}^{*}>0$, $\lambda _{2,k}^{*}=0$, we can obtain:

37 $$\begin{array}{*{20}l} m_{k}(a_{k} + b_{k}) < \frac{(a_{k}+b_{k})^{2}}{\lambda_{3}^{*}}. \end{array} $$

Then, we have:

38 $$\begin{array}{*{20}l} x_{k}^{*}=0. \end{array} $$

(c) If $x_{k}^{*}=d_{k}$, then $\lambda _{1,k}^{*}=0$, $\lambda _{2,k}^{*}>0$, we have:

39 $$\begin{array}{*{20}l} m_{k}(a_{k} + b_{k}) > \frac{(n_{k}d_{k}+a_{k}+b_{k})^{2}}{\lambda_{3}^{*}}. \end{array} $$

Then, $x_{k}^{*}$ is obtained as:

40 $$\begin{array}{*{20}l} x_{k}^{*}=d_{k}. \end{array} $$

Thus, the optimal power allocation solution can be given by:

41 $$ {}{\fontsize{7.6pt}{9.6pt}{x_{k}^{*}\,=\,\left\{\!\! \begin{array}{ll} 0, & m_{k}(a_{k} + b_{k}) \leq \frac{(a_{k} + b_{k})^{2}}{\lambda_{3}^{*}}, \\ -(\frac{a_{k}+b_{k}}{n_{k}}) \,+\, \frac{\sqrt{\lambda_{3}^{*}m_{k}(a_{k} + b_{k})}}{n_{k}},\!\!\! & \frac{(a_{k} + b_{k})^{2}}{\lambda_{3}^{*}} \!<\! m_{k}(a_{k} + b_{k}) \!<\! \frac{(n_{k}d_{k} + a_{k} + b_{k})^{2}}{\lambda_{3}^{*}},\\ d_{k}, & m_{k}(a_{k} + b_{k}) \geq \frac{(n_{k}d_{k} + a_{k} + b_{k})^{2}}{\lambda_{3}^{*}}, \end{array} \right.}}  $$

where $\lambda _{3}^{*}$ is the Lagrange dual variable corresponding to the constraint on the SINR performance:

42 $$ \sum\limits_{k=0}^{K-1} \frac{x_{k}^{*}m_{k}}{x_{k}^{*}n_{k} + a_{k} + b_{k}} \geq \text{SINR}_{\text{min}}.  $$

The iterative procedure of Criterion 2 is shown in Algorithm 2.



### LPI-based radar waveform optimization Criterion 3

In this case, if the communication signals scattered off the target are ignored at the radar receiver, we thus have *a*_*k*_=0. Then, the SINR expression is given by:

43 $$ \text{SINR} \triangleq \sum\limits_{k=0}^{K-1} \frac{x_{k}m_{k}}{x_{k}n_{k} + b_{k}}.  $$

Proceeding as before, we can write the optimization problem as follows:

44a $$\min\limits_{\{x_{k}\}}~\sum\limits_{k=0}^{K-1} x_{k},  $$

44b $$\begin{array}{*{20}l} &s.t.:\quad\left\{ \begin{array}{l} \sum_{k=0}^{K-1} \frac{x_{k}m_{k}}{x_{k}n_{k} + b_{k}} \geq \text{SINR}_{\text{min}}, \\ \mathbf{0} \leq \mathbf{x} \leq \mathbf{d}. \end{array} \right. \end{array} $$

We employ again the approach of Lagrange multipliers as in (17), we obtain the objective function:

45 $$\begin{array}{*{20}l} L(\mathbf{x}, \boldsymbol{\lambda}_{1}, \boldsymbol{\lambda}_{2},\lambda_{3}) & = \sum\limits_{k=0}^{K-1}x_{k}+ \boldsymbol{\lambda}_{1}^{T}(-\mathbf{x})+ \boldsymbol{\lambda}_{2}^{T}(\mathbf{x}-\mathbf{d}) \\ & + \lambda_{3} \left (\text{SINR}_{\text{min}} - \sum\limits_{k=0}^{K-1} \frac{x_{k} m_{k}}{x_{k} n_{k} + b_{k}} \right). \end{array} $$

Taking the first derivative of (45) with respect to *x*_*k*_, and setting it to zero, we can obtain:

46 $$\begin{array}{*{20}l} \frac{\partial L}{\partial x_{k}}=1-\lambda_{1,k}+\lambda_{2,k}-\lambda_{3} \cdot \frac{m_{k}b_{k}}{(n_{k}x_{k}+b_{k})^{2}}=0. \end{array} $$

After basic algebraic manipulations, we can obtain the optimal solution $x_{k}^{*}$ as a function of the Lagrange multipliers:

47 $$\begin{array}{*{20}l} x_{k}^{*}=-\frac{b_{k}}{n_{k}}+\frac{1}{n_{k}}\sqrt{\frac{\lambda_{3}^{*}m_{k}b_{k}}{1-\lambda_{1,k}^{*}+\lambda_{2,k}^{*}}}. \end{array} $$

From complementary slackness, we must consider the following three cases:

(a) If $0<x_{k}^{*}<d_{k}$, then $\lambda _{1,k}^{*}=\lambda _{2,k}^{*}=0$, we have:

48 $$\begin{array}{*{20}l} \frac{{b_{k}^{2}}}{\lambda_{3}^{*}} < m_{k}b_{k} < \frac{(n_{k}d_{k} + b_{k})^{2}}{\lambda_{3}^{*}}. \end{array} $$

Then, $x_{k}^{*}$ can be given as:

49 $$\begin{array}{*{20}l} x_{k}^{*}=-\frac{b_{k}}{n_{k}}+\frac{1}{n_{k}}\sqrt{\lambda_{3}^{*}m_{k}b_{k}}, \end{array} $$

where *λ*_3_ is a constant determined by:

50 $$\begin{array}{*{20}l} \sum\limits_{k=0}^{K-1} \frac{x_{k}^{*}m_{k}}{x_{k}^{*}n_{k} + b_{k}} \geq \text{SINR}_{\text{min}}. \end{array} $$

(b) If $x_{k}^{*}=0$, then $\lambda _{1,k}^{*}>0$, $\lambda _{2,k}^{*}=0$, we can obtain:

51 $$\begin{array}{*{20}l} m_{k}b_{k} < \frac{{b_{k}^{2}}}{\lambda_{3}^{*}}. \end{array} $$

Then, $x_{k}^{*}$ can be given as:

52 $$\begin{array}{*{20}l} x_{k}^{*}=0. \end{array} $$

(c) If $x_{k}^{*}=d_{k}$, then $\lambda _{1,k}^{*}=0$, $\lambda _{2,k}^{*}>0$, we can have:

53 $$\begin{array}{*{20}l} m_{k}b_{k} > \frac{(n_{k}d_{k}+b_{k})^{2}}{\lambda_{3}^{*}}. \end{array} $$

Then, $x_{k}^{*}$ can be given by:

54 $$\begin{array}{*{20}l} x_{k}^{*}=d_{k}. \end{array} $$

Finally, the optimal power allocation solution can be obtained as:

55 $$ x_{k}^{*}=\left\{ \begin{array}{ll} 0, & m_{k}b_{k} \leq \frac{{b_{k}^{2}}}{\lambda_{3}^{*}}, \\ -\frac{b_{k}}{n_{k}} + \frac{\sqrt{\lambda_{3}^{*}m_{k}b_{k}}}{n_{k}}, & \frac{{b_{k}^{2}}}{\lambda_{3}^{*}} < m_{k}b_{k} < \frac{(n_{k}d_{k} + b_{k})^{2}}{\lambda_{3}^{*}},\\ d_{k}, & m_{k}b_{k} \geq \frac{(n_{k}d_{k} + b_{k})^{2}}{\lambda_{3}^{*}}, \end{array}\right.  $$

where $\lambda _{3}^{*}$ is the Lagrange dual variable corresponding to the constraint on the SINR performance:

56 $$ \sum\limits_{k=0}^{K-1} \frac{x_{k}^{*}m_{k}}{x_{k}^{*}n_{k} + b_{k}} \geq \text{SINR}_{\text{min}}.  $$

The iterative procedure of Criterion 3 is detailed as follows.



*Remark 3:* The complexity of Algorithm 1 is dominated by the procedure of iterative search method and the number of subcarriers. In Algorithm 1, the complexity of the loop inside the step 2 is $\mathcal {O}(K)$. The convergence rate of the step 2 is based on the exhaustive search approach, which is given by $\mathcal {O}(\lambda _{3}^{*}/\epsilon)$. The total computational complexity of Algorithm 1 is $\mathcal {O}(K\lambda _{3}^{*}/\epsilon)$. Moreover, we can notice that the LPI based adaptive radar waveform optimization problems (14), (30), and (44) have a similar structure, which are different from each other in the way the cellular communication signals scattered off the target contribute to the signal part in (14), to the interference part in (30) and to neither in (44) [11]. Thus, Algorithms 2 and 3 have the same complexity as Algorithm 1, i.e., $\mathcal {O}(K\lambda _{3}^{*}/\epsilon)$.

*Remark 4:* Note that the communication signals scattered off the target would be an important component in target detection, which means that the energy corresponding to such scattering improves the detection performance of the radar system. While if the communication signals scattered off the target are not taken into account for the alternative hypothesis of the Neyman-Pearson (NP) detector, the detected energy would be reduced, resulting in a considerably lower target detection performance [10, 11]. In the following, it will be shown that the radar waveform optimization Criteria 2 and 3 offer inferior LPI performance for the radar system compared to the one that adopts Criterion 1 in the joint radar and cellular communication systems.

## Numerical results

In this section, we provide numerical results to demonstrate the enhancement of the LPI performance brought by our proposed adaptive radar waveform optimization criteria. Throughout the simulations, the carrier frequency of the joint radar and cellular communication systems is 3 GHz. We set the total bandwidth to be 512 MHz, which is equally divided by *K*=128 subcarriers. A total of *N*_*t*_=3 communication systems are operating from channel 1 to 40, 41 to 80 and 81 to 128. In order for the cellular communication systems to function properly, the achievable capacity for each channel must be above a predetermined threshold, which is set to be *t*_*k*_=1.0 nat/symbol(∀*k*). The system parameters are given as shown in Table 1.

**Table 1:** System parameters

Parameter	Value	Parameter	Value
*G*_*t*_	30 dB	*G*_*s*_	0 dB
*d*_*r*_	12 km	*d*_*s*,*i*_(∀*i*)	8 km
*d*_*t*,*i*_(∀*i*)	1 km	*d*_*b*,*i*_(∀*i*)	20 km
*P*_max,*k*_	200 W	SINR_min_	10 dB
*λ*_*k*_(∀*k*)	0.10 m	${\sigma _{n}^{2}}[k] (\forall k)$	1.66×10^−14^ W

**Fig. 2:**
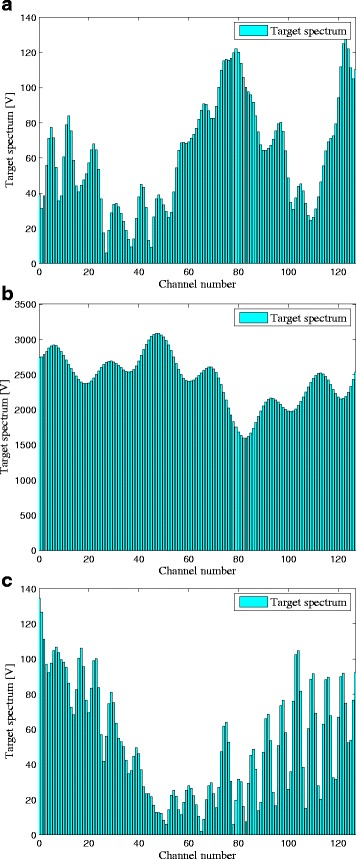
The target spectra of corresponding channels: **a** *H*_*r*_[*k*]; **b** *H*_*s*_[*k*]; **c** *H*_*e*_[*k*]

**Fig. 3:**
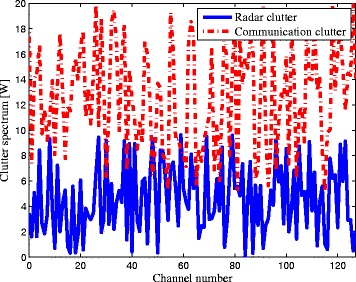
Clutter spectra

**Fig. 4:**
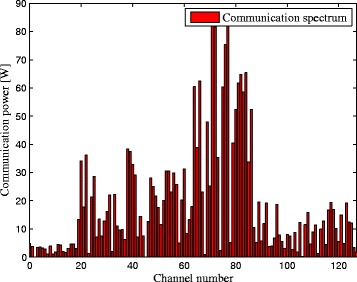
Communication transmit power

Herein, we consider a scenario that the stealth target is illuminated from the front by the radar signals and from the side by the cellular communication signals. Hence, the communication signal scattered off the target would be a much more important component in target detection than the scattered radar signal. The target spectra of the corresponding channels *H*_*r*_[*k*], *H*_*s*_[*k*], and *H*_*e*_[*k*] are shown in Fig. 2 respectively, which shows the target spectral impulse responses for each subcarrier. In a realistic case, the target spectra can be provided by the target database based on the target-radar/BSs orientations. The PSDs of the signal-dependent clutters due to the radar and communication BS transmitted signals are illustrated in Fig. 3, which can be obtained by sensing with a spectrum analyzer or prior knowledge. It can be clearly observed from Fig. 3 that the PSD values of clutters for each subcarrier are shown. Figure 4 gives the communication transmit power provided by the cellular communication systems. The red bars represent the transmit power of the communication systems in each channel. Given the exact characteristics of the communication signals, the target spectra, the clutter PSDs, and the propagation losses of corresponding channels, we can obtain the optimal solutions of problems (14), (30), and (44).

**Fig. 5:**
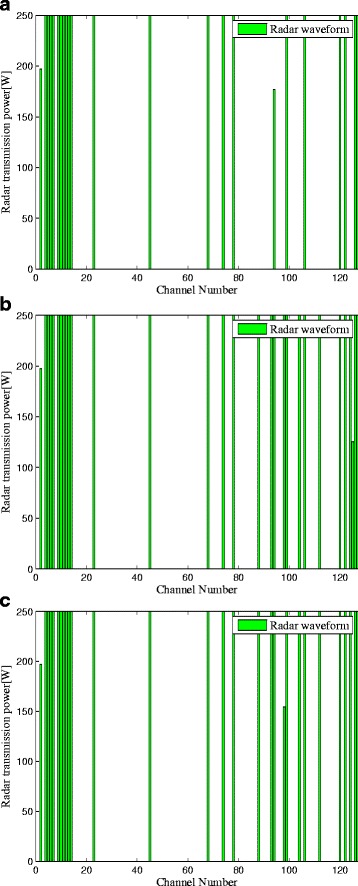
LPI based radar waveform design results: **a** Criterion 1; **b** Criterion 2; **c** Criterion 3

Figure 5 depicts the LPI-based radar waveform design results, which gives insight about the optimal power allocation for LPI performance in the radar system. For all the criteria presented here, more transmission power is allocated to the channels which have larger gain of *H*_*r*_[*k*] and suffer less interference power provided by the cellular communication systems. Specifically, to minimize the total transmitting power for a predefined SINR constraint and a minimum required capacity for the communications systems, the LPI-based transmission waveform criteria are formed by water-filling, which only places the minimum power over the dominant frequency components of the channel, that is, the subcarriers with the largest gain and least interference power [24].

Moreover, it is apparent that more transmit power is placed between the subcarrier 80 and 128 in Criteria 2 and 3 than that in Criterion 1. The results show that the total transmitted power of Criterion 1 is 5.6242 KW, while the transmitted power of Criteria 2 and 3 is 7.3223 and 7.1017 KW, respectively. It indicates that the LPI performance of Criterion 1 outperforms that of Criterion 2 by 30.19 *%* and that of Criterion 3 by 26.27 *%*, which is due to the fact that the communication signals scattered off the target would be a much more significant component in target detection than the radar signals [10, 11]. That is to say, if the communication waveform scattered off the target is not considered for target detection, the detected energy is reduced. We can conclude from Figs. 2, 3, 4, and 5 by stating that the LPI performance of the radar system benefits significantly from taking into consideration the communication signals scattered off the target at the radar receiver.

**Fig. 6:**
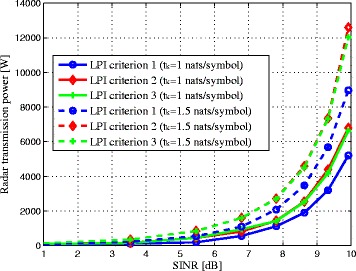
Radar transmission power of the radar system versus SINR with different *t*_*k*_ utilizing optimal waveform optimization criteria

Figure 6 depicts the radar transmission power of the radar system versus SINR with different *t*_*k*_ employing the proposed optimal waveform optimization criteria, which are conducted 1000 Monte Carlo trials. It can be seen that the transmission power increases as the value of SINR goes up. In addition, one can observe that, as the required channel capacity for the communication system increases, more transmission power will be transmitted to satisfy the predetermined target detection performance of a radar system. Physically speaking, when the capacity threshold for each channel increases, *c*_*k*_ is then reduced. Thus, there will be two power levels either *d*_*k*_ when there is transmission or zero otherwise. As a consequence, more channels will be allocated the maximum power *d*_*k*_ to guarantee the given SINR requirement.

**Fig. 7:**
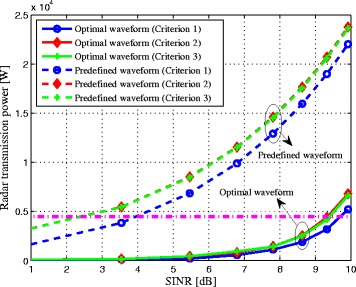
Radar transmission power of the radar system versus SINR in each Criterion with different waveform optimization methods

It is shown in Fig. 7, the radar transmission power versus SINR in each Criterion with different waveform optimization methods. As expected, the best achievable SINR can be obtained when utilizing the LPI-based optimal waveform optimization Criterion 1 with the same transmit power (see the pink dash-dot line), since the radar can make use of the communication signals scattered off the target for a better target detection capability [11]. Thus, it will in turn transmit the minimum power for a given threshold of SINR and offer the best LPI performance for the radar system. On the other hand, the radar waveform that employs Criterion 2 offers a slightly lower LPI performance than the waveform that uses Criterion 3. The reason is that Criterion 3 ignores the scattering off the target due to the communication signals rather than considers it as interference. The transmission power of the predefined waveform is also plotted, which allocates the transmission power uniformly in the whole frequency band without any prior knowledge of the target spectra, the clutter PSDs, and the communication signals. We can notice that the predefined waveform exhibits a much worse LPI performance than that of the optimal waveform. Overall, the presented LPI-based radar waveform design criteria give insight about the optimal power allocation. These three techniques are easy to realize and will undoubtedly achieve much better LPI performance by exploiting the scattering off the target due to communication signals.

## Conclusions

In this paper, we have studied the problem of LPI-based radar waveform optimization in signal-dependent clutter for joint radar and cellular communication systems, where three different LPI based criteria are presented for radar waveform optimization. For each criterion, the total transmitted power of the radar system is minimized and the associated optimization problem is formulated and solved analytically. Using numerical results, we have proven that the LPI performance of radar system can be considerably enhanced by exploiting the communication signals scattered off the target at the radar receiver. Future work will investigate the LPI-based robust radar waveform design for joint radar and cellular communication systems.
